# A New Strategy of Lithography Based on Phase Separation of Polymer Blends

**DOI:** 10.1038/srep15947

**Published:** 2015-10-30

**Authors:** Xu Guo, Long Liu, Zhe Zhuang, Xin Chen, Mengyang Ni, Yang Li, Yushuang Cui, Peng Zhan, Changsheng Yuan, Haixiong Ge, Zhenlin Wang, Yanfeng Chen

**Affiliations:** 1 Department of Materials Science and Engineering, College of Engineering and Applied Sciences, Nanjing University, Nanjing, 210093, China; 2 Department of Physics, Nanjing University, Nanjing, 210093, China; 3 National Laboratory of Solid State Microstructures, Nanjing, 210093, China; 4 Jiangsu Provincial Key Laboratory of Advanced Photonic and Electronic Materials, School of Electronic Science and Engineering, Nanjing University, Nanjing, 210093, China; 5 Department of Polymer Science & Engineering and Key Laboratory of High Performance Polymer Materials & Technology of MOE, School of Chemistry & Chemical Engineering, Nanjing University, Nanjing, 210093, China; 6 Collaborative Innovation Center of Advanced Microstructures, Nanjing, 210093, China

## Abstract

Herein, we propose a new strategy of maskless lithographic approach to fabricate micro/nano-porous structures by phase separation of polystyrene (PS)/Polyethylene glycol (PEG) immiscible polymer blend. Its simple process only involves a spin coating of polymer blend followed by a development with deionized water rinse to remove PEG moiety, which provides an extremely facile, low-cost, easily accessible nanofabrication method to obtain the porous structures with wafer-scale. By controlling the weight ratio of PS/PEG polymer blend, its concentration and the spin-coating speed, the structural parameters of the porous nanostructure could be effectively tuned. These micro/nano porous structures could be converted into versatile functional nanostructures in combination with follow-up conventional chemical and physical nanofabrication techniques. As demonstrations of perceived potential applications using our developed phase separation lithography, we fabricate wafer-scale pure dielectric (silicon)-based two-dimensional nanostructures with high broadband absorption on silicon wafers due to their great light trapping ability, which could be expected for promising applications in the fields of photovoltaic devices and thermal emitters with very good performances, and Ag nanodot arrays which possess a surface enhanced Raman scattering (SERS) enhancement factor up to 1.64 × 10^8^ with high uniformity across over an entire wafer.

Fabrication of nanostructures on aiming substrates is playing an unprecedentedly important role in fundamental sciences and applied technologies. Generally, there are two major approaches to fabricate nanostructures categorized as top-down and bottom-up method^1^,^2^. Top-down approaches offer the advantages of high fidelity and high controllability. Among them, photolithography has been well developed and widely used by conventional semiconductor industry, however the feature size is strongly restricted by the optical diffraction limit^3^. Ultra-small structural units with very fine structures could be fabricated via e-beam lithography and ion beam lithography, while these techniques have very low throughput and might be high-cost^4^. In addition, photolithography^3^, nanoimprint lithography^5^ and soft lithography^6^ usually require a prefabricated mask or mold. As alternative pathways, there have been growing interests in the formation of nanostructures by bottom-up approaches. Compared with top-down methods, bottom-up methods provide low-cost and simple processes for nanofabrication, while it is prone to unavoidable defects and lack of degree on pattern control. Typical strategy of bottom-up approaches is the self-assembly of amphiphilic lipids^7^, surfactants^8^, block-copolymers^9^, polymer blends^10^ or colloidal particles^11^ into various patterns and periodicities. Among them, phase separation has drawn intensive attentions over the past two decades and shows great potential in fabrication of optical and electronic devices^12^, energy storage^13^, catalyst supports^14^, templates^15^,^16^, cell culture scaffolds^17^, and super hydrophobic surfaces^18^. Phase separation systems include block copolymers^19^,^20^, polymer blends^21^,^22^ and breath figures (BF)^16^,^23^,^24^, and it has been reported that self-assembly of block copolymer with a suitable choice of immiscible segments and their chain lengths can achieve well-ordered nanostructures down to sub–20 nm scale on large areas^25^. These well-ordered nanostructures have been successfully applied in fabrication of porous membranes with controlled nanostructures^26^, photonic crystals^27^,^28^, metallic and oxide nanodot arrays with high resolution^29^,^30^, and even masks for follow-up nanolithography^25^,^31^. However, besides the complicated synthetic routes of block copolymers and the long subsequent processing time for phase separation, the immiscible segments are covalently bonded to each other which causes that relief patterns cannot be directly formed by selectively removing one block of the copolymer with a solvent as the development process for photolithography. Recently, phase separation of polymer blends or the water with the polymers in breath figure approaches has been studied to allows the production of complex layered or lateral nanostructures in a simple and low-cost one step way, especially through a spin coating process, which could be used to form semiconducting polymer-based devices, such as light-emitting diodes and photovoltaics^32^,^33^.

Although now available lithographic techniques have almost reached perfection, the formation of nanostructures in an inexpensive and facile way is continuously very attractive and important both in research and production. So far, most studies on phase separation of polymer blends or breath figures have been restricted to formation of the final polymers micro- and nanostructures. There are few studies to employ the phase separation of polymer blends as a lithographic technique to directly pattern aiming substrates in combination with follow-up developments such as metal evaporation, lift-off and reactive ion etching (RIE) techniques. In this paper, we present a simple and effective maskless lithographic approach based on phase separation of polystyrene (PS) and polyethylene glycol (PEG) immiscible polymer blend system. Its simple process only involves spin-coating the polymer blend solution, followed by deionized water rinse. In control of solvent type, PS/PEG ratio and concentration, and spin-coating speed, the formation of a single layer of isolated and elevated PEG droplets with controllable size dispersed in continuous PS phase can take place exclusively with wafer-scale. By selective dissolution of the PEG moiety with water, porous nanostructures are left on the surface of PS thin-film, which is quite similar to the development process of photolithography. The approach is compatible with existing nanofabrication techniques to further transfer the porous nanostructures to other substrates. This simple process allows us to fabricate micro/nanostructures with controllable structural parameters in a convenient way. To illustrate its potential applications, we fabricate wafer-scale anti-reflection (AR) structures on the upper surface of silicon wafers and Ag dot arrays for surface enhanced Raman scattering (SERS), respectively. The AR silicon nanostructures exhibit an outstanding quality with a reflectance below 3% over a broad spectrum wavelength regime from 450 to 950 nm. The Ag dot arrays on silicon substrate possess a SERS enhancement factor up to 1.64 × 10^8^ with high uniformity across over an entire 4″ wafer. The micro/nanostructures fabricated by this new strategy of lithography show great reproducibility of physical and chemical properties in wafer-scale. Also, this maskless lithography will meet the requirements of industrial production due to its high-throughput and low-cost advantages.

## Results

### Phase separation lithography based on PS and PEG blends

**Figure 1:**
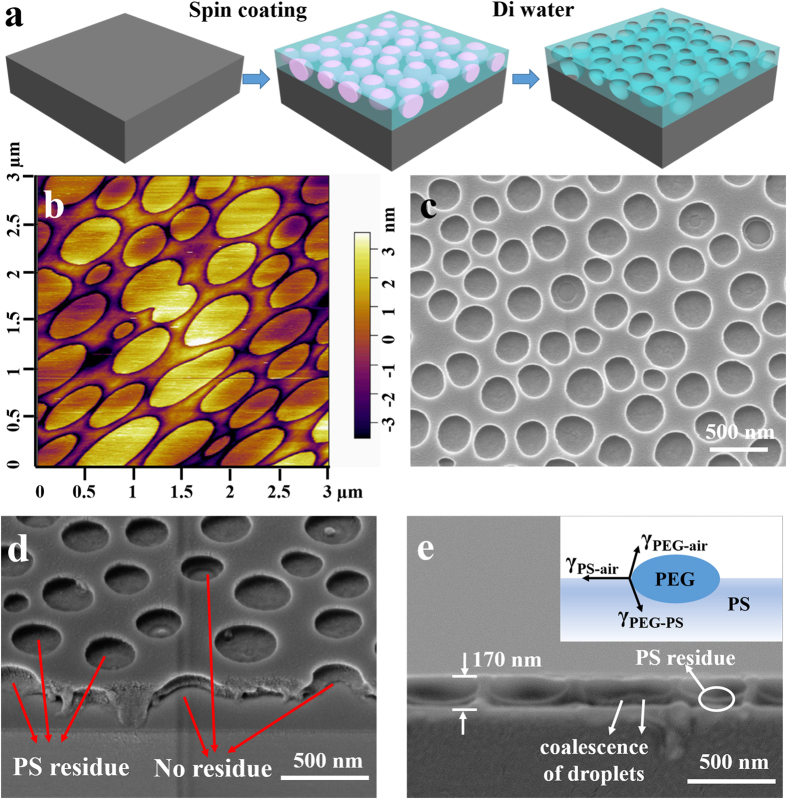
(**a**) Schematic of phase separation of PS and PEG polymer blends. (**b**) AFM image of spin-coated PS/PEG blend film. (**c**) Top SEM view and (**d**) tilted SEM view of PS nanopore structures after PEG removal. (**e**) Cross-sectional view of SEM image of PS nanopore structures with a height of 170 nm and a residual layer of ~30 nm and the inset of the schematic illustration of PEG droplet at the air/PS phase interface. The polymer blend concentration was 5 wt% with the ratio of PS:PEG = 2:3 (w/w) and the spin speed was set at 3000 r∙min^−1^.

The PS/PEG blends are typical phase separation systems which have been used to prepare periodic nanoporous films through solvent evaporation^34^. In this work, PS/PEG blends are chosen as the base of the lithographic resist materials. Figure 1a schematically presents the procedure of the phase separation lithography. In brief, solutions of PS/PEG polymer blends were prepared by dissolving PS and PEG into toluene with various weight ratios of PS/PEG. The PS/PEG blend film was spin-coated on a silicon substrate, followed by rinse with deionized (DI) water. Due to its good water-solubility, the PEG component of the polymer blend films could be thoroughly removed by water without deteriorating the remained PS structures. This step is very similar to the development process of photolithography. After blow drying the sample with nitrogen gas, the PS nanoporous network would be obtained. Figure 1b shows the atomic force microscope (AFM) image of the spin-coated PS/PEG blend film with a PS/PEG weight ratio of 2:3 and 5 wt % toluene solution at a spin speed of 3000 r∙min^−1^, which clearly indicates the phase separation morphology. The film surface was characterized by a great number of isolated small islands distributed randomly in a continuous matrix and the isolated islands slightly protruded (about 6 nm higher) from the continuous background. The assignment of respective polymers to the different domains was easily determined by phase separation process of the polymer blend and the following selective dissolution of the PEG in water. The scanning electron microscopy (SEM) image (Fig. 1c) of the same sample in Fig. 1b after water rinse exhibits a porous nanostructure, which indicates that the continuous film was composed of PS and the isolated islands were PEG domains because the PEG could by selectively dissolved in water. Compared with the nearly circular nanopore shape in the SEM image, the AFM image of the PEG islands was elongated in the direction of 45^o^ relative to the scanning direction, which may be caused by the viscous force between the AFM tip and the liquid PEG domain. In addition to circular shape, some elliptical and deformed pores are also observed from the top-view SEM image. The diameter of the pores mainly ranges from about 200 to 400 nm and the distance between every two nanopores is about 100 nm. The tilted SEM image (Fig. 1d) shows that some pores are completely perforated and the others still have a residual PS layer attached on the substrate. The cross-sections of the pores (Fig. 1e) exhibited ellipsoidal shapes. The PS multiporous film is homogenous with thickness of 170 nm, and continuous PS layer under the bottom of the pores is less than 30 nm thick.

The experimental results shown in Fig. 1 allow us to draw a phase separation sketch of ellipsoidal PEG droplets laterally distributed in the continuous PS matrix. The main stages of phase separation could be recognized as following. In the first stage, the polymer blend solution was spread out to cover the substrate and spun off the edge of substrate by centrifugal force, and a thin film of polymer solution was left on the substrate. Then, this solution film continuously thinned with a radial liquid flow and simultaneous evaporation of solvent. During this stage, due to the lower solubility of PEG in toluene, the PEG phase first precipitated from the solution with the increase of the blend concentration as the solvent (toluene) evaporated. The surface tension effect drove the PEG domain to form isolated droplets dispersed in PS/toluene matrix. As time proceeded, the small droplets coalesced resulting in larger droplets. The elliptical or deformed pore shape as shown in Fig. 1c could be attributed to the coalescence of PEG droplets in polymer blend. This mechanism was confirmed by the cross-sectional SEM image (Fig. 1e). It is clearly seen that the coalescing of two PEG droplets was not terminated when the PS phase was solidified. Finally, the evolution of overall phase structures was frozen when the toluene solvent was depleted. If the vertical scale of the PEG domain was close to the final PS film thickness which could be precisely controlled by spin-coating process, the resultant nanopore structures could exclusively locate in plane of the film. In addition, the vertical ellipsoidal nanopore shape was led by the interaction of the surface tensions of each polymer phase and interfacial tension between PEG and PS phase as the inset of the schematic illustration in Fig. 1e^23^.

**Figure 2:**
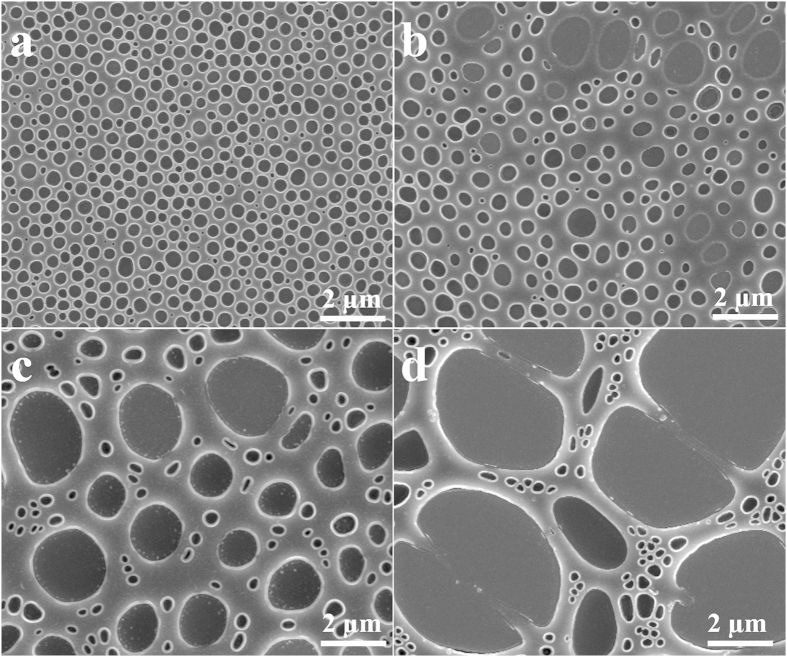
Top view of SEM images of porous PS film with different PS/PEG weight ratio. The initial polymer blend concentration was set at 5 wt% and the spin speed was 4000 r min^-1^. (**a**) PS:PEG = 1:2; (**b**) PS:PEG = 1:2.5; (**c**) PS:PEG = 1:3; (**d**) PS:PEG = 1:4.

The effects of the weight ratio of the PS/PEG blend, concentration of PS/PEG blend in toluene and spin speed on the geometry properties of porous nanostructure were investigated. Four polymer blend solutions with the PS/PEG weight ratios of 1:2, 1:2.5, 1:3 and 1:4, were prepared, while the initial concentration of the solution of PS/PEG polymer blend in toluene was set at 5 wt %. Figure 2 shows SEM images to present the typical geometries of the film surfaces which are formed with different PS/PEG weight ratios after water rinsing. In order to understand the variation trend visually, the feature size distributions of such PS nanopores are drawn according to the SEM images, as shown in Fig. S1. The average pore size, its variance, and the number density of pores dependent on different PS/PEG weight ratios were measured and counted from the corresponding larger areas of the samples as shown in Table 1. The pore size and pore area increases along with the increase of PEG fraction in the blend, while the uniformity of pore size decreased sharply. It is obvious that two types of the pores with quite different sizes could be clearly seen from the films prepared by blend polymer with larger PEG ratio as shown in Fig. 2c,d in which larger pores with dimensions of several microns randomly distributed in the PS matrix surrounded by a great number of small (several hundred nanometers in size) pores. The larger pores were formed from the coalescence of the small PEG droplets. Coalescence of the PEG droplets was controlled by the viscosity of the continuous PS phase, which is mainly led by the PS concentration in toluene phase. The higher viscosity of the PS in toluene phase could suppress the coalescence of the PEG droplets after the phase separation since floating of these droplets were somehow restricted in the continuous phase. A reduction of PS fraction in the polymer blend solution would decrease the viscosity of the PS in toluene phase, which means the pore size would increase with the relative increase of PEG fraction in the polymer blend. In addition to the pore structure, a few of particle shape domains were observed in the pores as shown in SEM images of Figs 1 and 2, which could be attributed to the secondary phase separation occurring in the precipitated PEG domains. As the PEG-rich phase was separated from the toluene solution, a small fraction of PS remained in the PEG-rich phase was inevitable, and these PS might be further emulsified and precipitated to form small PS particles in each PEG droplet with the toluene evaporation.

**Figure 3:**
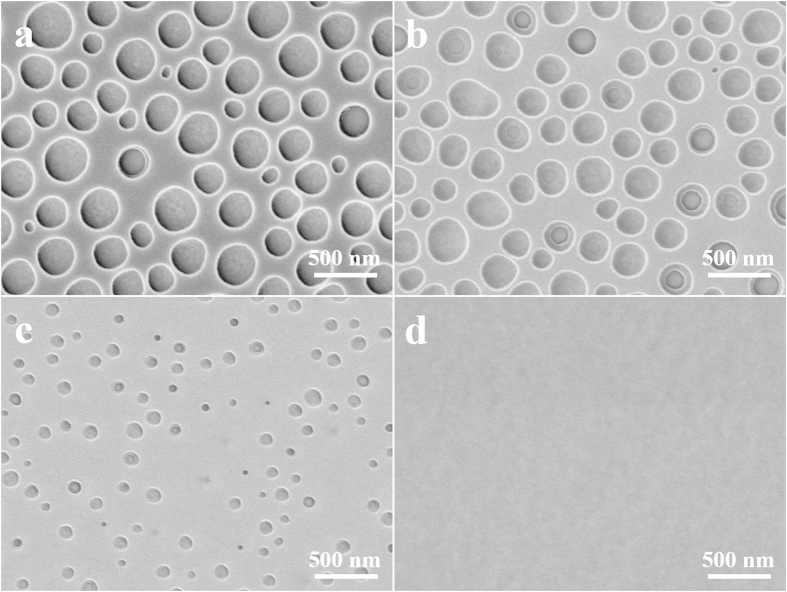
Top view of SEM images of PS porous film with different polymer blend concentration. (**a**) 5 wt%; (**b**) 4 wt%; (**c**) 3 wt% and (**d**) 2 wt%. The initial PS/PEG weight ratio was set at 2:3 and the spin speed was 3000 r∙min^–1^.

Figure 3 shows the phase separation results of PS/PEG solutions with the concentrations of 5, 4, 3 and 2 wt %, while the initial PS/PEG weight ratio was fixed at 2:3. In the same way, the feature size distributions of PS nanopores are drawn according to the corresponding SEM images so as to understand the variation trend visually, as shown in Fig. S2. The pore size and total pore area drastically decreased with reducing solution concentration. It was almost hard to find any nanopore structures on the water-rinsed film as the concentration decreased to 2 wt %. The average pore size and pore density were summarized in Table 1. As a matter of fact, the reduction of the PS/PEG blend concentration also decreased the amount of PEG in solution such that the precipitation of PEG phase from the toluene was hard to occur until a higher PEG concentration was obtained as the toluene kept evaporating during the spin-coating process. Namely, less PEG was precipitated for the lower concentration solution when the PS matrix was solidified, which resulted in the nanopore structures with smaller pore size and lower total pore area.

Moreover, the spin speed of the spin-coating could also affect the morphology of nanopore led by phase separation of PS/PEG blends in toluene. Figure S3 a to c shows the SEM images of phase separation of PS/PEG with various spin speeds under the constant PS/PEG weight ratio of 1:2 and fixed solution concentration of 5 wt %. The size distributions of PS nanopores are drawn according to the SEM images and shown in Fig. S3 a’ to c’, respectively. With the increase of spin speed, the feature size of the nanopore gradually decreased (i.e. the diameter ranges from 102 to 727 nm for the spin speed at 3000 r∙min^−1^, while from 90 to 550 nm for 4000 r∙min^−1^ and from 84 to 501 nm for 5000 r∙min^−1^), and according to statistics, the discrepancies of the diameters of the nanopores became smaller, which is 95 nm for 3000 r∙min^−1^, 77 nm for 4000 r∙min^−1^ and 73 nm for 5000 r∙min^−1^, respectively, as summarized in Table 1. The solvent of PS/PEG solution volatilized more quickly under the higher spin speed so that there was not enough time for the dispersive PEG droplets to coalesce into larger droplets, which led to the diameter of pores smaller.

**Figure 4:**
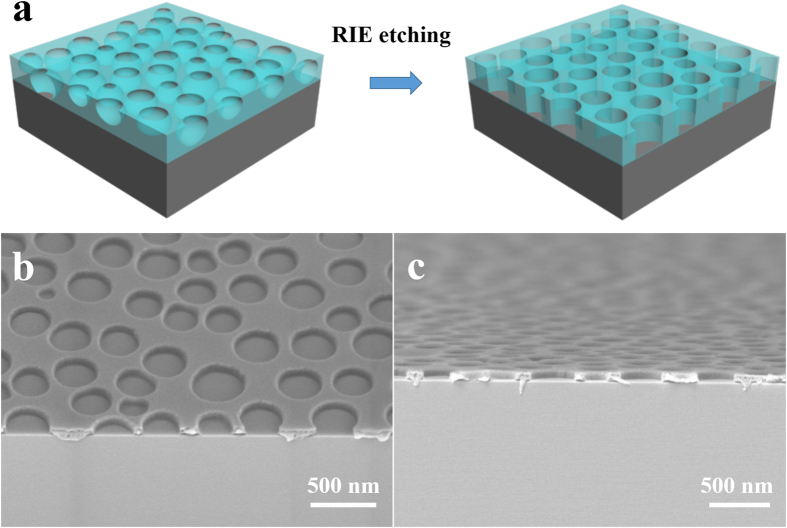
(**a**) Schematic of PS residue etching by O_2_ RIE process. (**b**) Tilted and (**c**) cross-sectional view of SEM images of PS porous film after PS residual was removed.

From the SEM images of the phase-separated films, we can see that there is still some residual PS at the bottom of the nanopore structures with thickness about 30 nm, which is necessary to be removed before this multi-porous microstructure could be used as a mask or template for further nano- and micro-fabrication processes. As shown in Fig. 4, the underlying residual PS thin-layer could be completely removed and the supporting silicon substrate was exposed through the nanopore after an O_2_ RIE process. The RIE-treated nanopore structures show smooth, straight and vertical sidewalls, which could be further used as sacrificial layer for lift-off process or as etching mask for pattern transfer.

### Applications of phase separation lithography

Although nanopore structures fabricated by the phase separation lithography is less uniform than those fabricated by self-assembly of block copolymers and traditional top-down methods, the feature size of the nanopore and the number density of pores can be almost the same as the ones made by top-down methods. More importantly, since this method is extremely simple, low-cost and easily accessible with wafer-scale, it is quite suitable to fabricate optical and optoelectronic devices that are dependent on the average effect of a large ensemble of all functional elements and allow some defect tolerance without losing any performance, such as solar cells^35^, displays^36^, optical sensors^37^, nano-patterned sapphire substrates for light-emitting diode (LED)^38^ and SERS substrates^39^. Aiming at different purposes, the required feature size of the nanostructures could be determined and controlled conveniently by tuning the PS/PEG concentration, the corresponding blend polymer weight ratio and the spin speed during the phase separation lithographic procedure.

**Figure 5:**
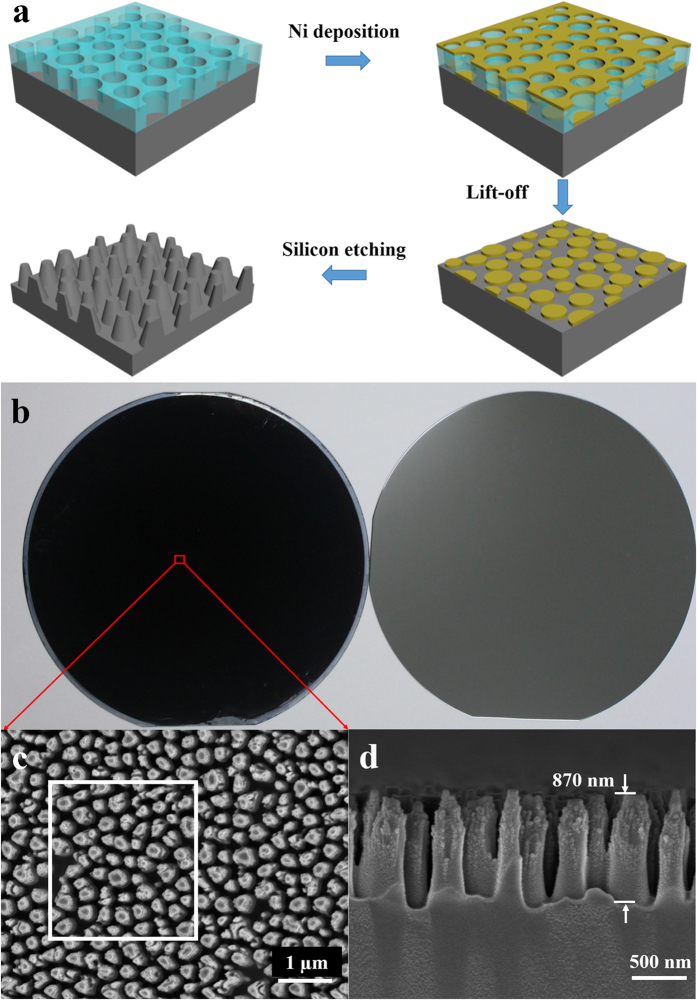
(**a**) Schematic of fabricating wafer-scale surface-modified silicon nanostructures for AR by spin-coating phase separation lithography. (**b**) Photographs of 4″ AR silicon wafer (left) and bare silicon wafer (right). (**c**) Top view and (**d**) cross-sectional view of SEM images of surface-modified silicon nanostructures.

In order to present the capability and superiority of phase separation lithography, we firstly demonstrated the fabrication of surface-modified silicon nanostructures with wafer-scale for AR through this method. The fabrication procedure is schematically shown in Fig. 5a. The PS nanopores on a 4″ bare silicon wafer was prepared by the above-mentioned phase separation lithography (details shown in the *experimental methods*), followed by oxygen-RIE process to remove the residual PS layer and to modify the PS nanopores with sharp, straight and vertical sidewalls. Afterwards, a 10 nm-thick nickel (Ni) layer was deposited on this pre-treated PS nanopore structures via an e-beam evaporation deposition, and then Ni nanodot arrays were left on the silicon substrate after a lift-off process in chlorobenzene. Finally, the silicon nanopillars array was achieved by RIE with CHF_3_ gas using the Ni nanodots as etching mask and the surplus nickel was removed by HNO_3_. Figure 5b shows the photograph of 4″ silicon wafer patterned by silicon nanopillars array (left), which looks totally black in color in comparison with optical photograph of a bare silicon wafer (right). Figure 5c,d shows the top and cross-sectional view SEM images of the as-prepared silicon nanopillars array, respectively, in which each silicon nanopillar exhibits a tapered shape with the height around 870 nm and the diameter below 500 nm.

**Figure 6:**
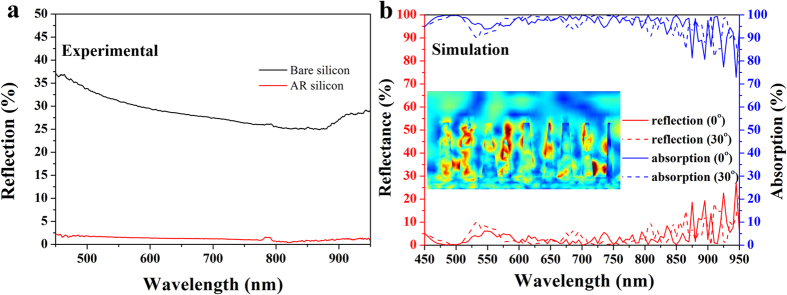
(**a**) Reflectance spectra of bare silicon wafer and AR wafer from 450 to 950 nm. The reflectance of AR wafer is under 3%. (**b**) Simulated optical reflection and absorption spectra of AR wafer with the incident angle of 0^°^ and 30^°^, respectively, and the inset shows the typical electric field distribution at the wavelength of 730 nm.

The as-prepared array of vertically aligned silicon nanostructures composed of taper-shaped Si nanopillars with high-aspect ratios shows near-zero reflection over a wide range of incident angles and a broad spectral bandwidth, since the silicon-based nanostructure that we fabricated acts as a membrane with a continuous refractive index gradient which might reduce Fresnel reflection^35^. The measured reflection spectra of the samples were obtained by an UV-VIS spectrophotometer with an integrating sphere at near-normal incident angle of 8°. Clearly shown as the red-line in Fig. 6a, for the sample of silicon wafer patterned with nanopillars array, the average reflectance is under 3% in a broad wavelength regime from 450 to 950 nm, which is drastically low compared to that (black line in Fig. 6a) for the bare silicon wafer.

To well demonstrate optical response of the silicon AR nanostructure, three-dimensional numerical simulations were performed using commercial software package (COMSOL Multiphysics) based on finite-element method. The simulation domain corresponds to actual region of the sample outlined with white box in SEM image of Fig. 5d, and periodic boundary conditions are applied to the four sides of the rectangular simulation domain to mimic the infinitely large area of the sample in the *xy*-plane. The refractive index of the silicon (*n*_*Si*_) is taken from Green’s research^40^, and the taper-shaped Si-nanopillars with the same height of 870 nm are vertical aligned on a very thick silicon base. In the simulation, a plane wave with linear polarization is illuminated on the surface-modified Si-nanostructures. In Fig. 6b, the simulated optical reflection spectra with the incident angle of 0^o^ (Red-solid line) and 30^o^ (Red-dashed line) were plotted, which indicates an excellent incident angle-independent anti-reflection covering a broad wavelength regime of visible and near-infrared. Overall, the simulation is in good agreement with the experimental observation. Considering optical opaque nature of this silicon nanostructures, the transmission (*T*) of the sample is neglected, thus the absorption (*A*) could be defined as *A* = *1–R* as plotted with Blue lines in Fig. 6b. As an example, we draw a map of the electric field distribution at wavelength of 730 nm as shown in the inset of Fig. 6b, and find that considerable portion of energy is localized in the regions of silicon nanopilliars, which indicates that this non-reflection silicon nanostructures could provide beneficial light trapping that eventually increases the effective length of light-matter interaction in the silicon. In addition, attributed to the random morphology of the nanostructure, the polarization-insensitive light reflection (absorption) is also expected (Results not shown here). The method based on phase separation lithography to fabricate surface-modified silicon nanostructures with wafer-scale is fairly simple, low-cost and tunable to structural parameters as well, and it is compatible with semiconductor device fabrication techniques such as RIE and physical vapor deposition (PVD), which might have potential applications in solar cells and other electro-optical devices.

**Figure 7:**
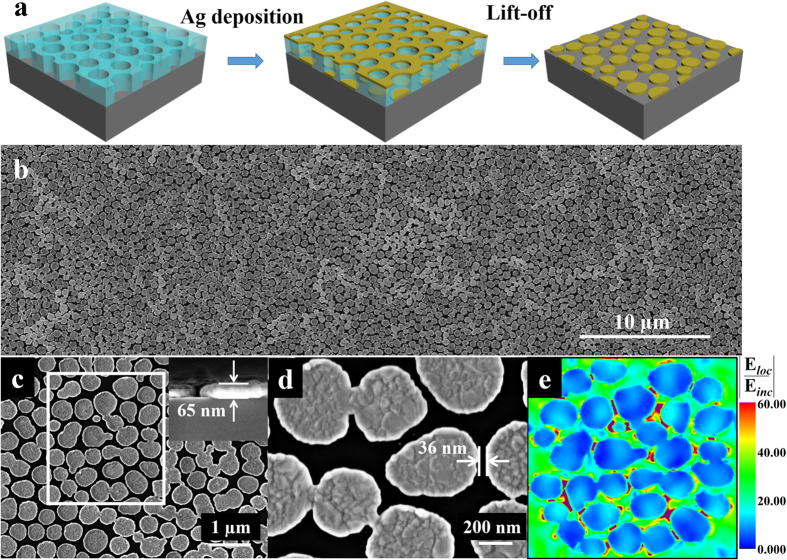
(**a**) Schematic of fabricating SERS-active substrate by the lithography process based on phase separation method. (**b–d**) present the top view of SEM images of Ag nanodot arrays with different magnification, respectively. The inset of (**c**) is the cross-sectional view of SEM image of a typical Ag nanodot with thickness of 65 nm. The distance between every two Ag nanodots is about 36 nm. (**e**) The calculated distribution of enhancement ratio of local electric field 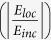 under the laser excitation with wavelength of 532 nm. The purple area stands for the enhancement ratios of local electric field which are greater than 60.

Metallic nanoparticle arrays have recently attracted considerable attention due to their plasmonic properties leading to giant enhancement of the local electric fields^41^,^42^,^43^, which enable them as a powerful platform for various applications in the fields of enhanced photoelectric conversion^44^, photocatalysis^45^, and detection of extremely weak spectral signals such as Raman signal and single-molecular fluorescence. By properly engineering the morphology and size of metallic nanostructures, the localized electric fields are concentrated and significantly enhanced at specific locations of the nanostructure surfaces to form the so-called “hot-spot”. This enhanced electric field can dramatically improve Raman signals, named as SERS. To date, various types of metallic nanostructures have been designed and fabricated as SERS substrates, even for single molecular detection^46^,^47^. Nevertheless, from a perspective of practical application, an ideal SERS-active chip should have dense packing of metal nanostructures, repeatable signal levels, economical construction and robustness to sustain sensing performance over the whole chip. Here we purpose a new strategy to prepared wafer-scale SERS-active chip via our developed phase separation lithography as schematically shown in Fig. 7a. A 65 nm thick Ag layer was deposited on the pre-treated PS nano-porous membrane by an e-beam evaporation coating, and then Ag dot arrays on Si wafer were obtained after a lift-off process in chlorobenzene. Figure 7b shows the top view of SEM image of Ag nanodots arrays with a large area and few defects. Figure 7c,d and the inset of Fig. 7c present the SEM images of Ag dot arrays from top and cross-sectional with enlarged view. Noted that the nanogaps between every two Ag particles might provide “hot-spots” to further enhanced localized electric field due to the coupling of plasmon resonances of individual Ag particles, which has been manifested by the numerical simulation.

**Figure 8:**
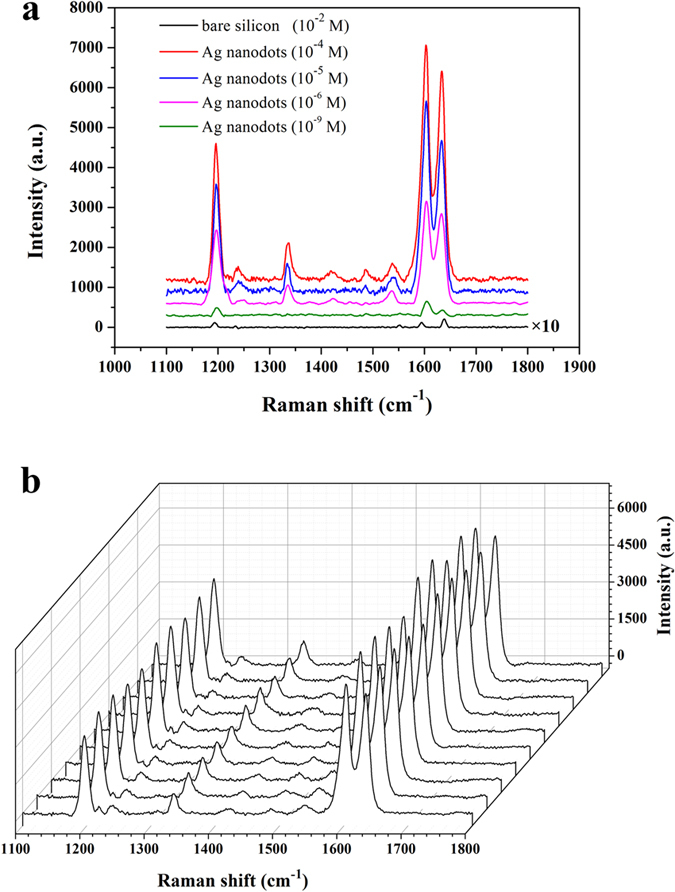
(**a**) Raman shift of BPE (10^−4^, 10^−5^, 10^−6^ and 10^−9^ M) on SERS substrate of Ag nanodots and bare silicon wafer (10^−2^ M) (exposure time = 10 s). The Raman intensity of BPE on bare silicon was multiplied by 10. (**b**) Reproducibility test for SERS spectra of BPE molecules at ten random different points on the SERS substrate of Ag dot arrays. (BPE = 10^−4^ M; exposure time = 10 s).

The simulation region was selected according to the SEM image of sample, shown as the white box in Fig. 7b. Silver is described as a dispersive medium with the complex dielectric parameters taken from experimental data by Johnson and Christy^48^. Figure 7e plotted the electric field distribution under the laser excitation with wavelength of 532 nm, and it is clear that the electric field is dramatically enhanced in the gap area between the Ag nanoparticles (the purple area stands for the enhancement ratios of local electric field which are greater than 60), which stems from the near-field coupling of the plasmon resonance of individual nanoparticle. Figure 8a shows the Raman spectra of trans-1,2-bis(4-pyridyl)ethylene (BPE) on bare silicon and Ag dot arrays at the concentration of 10^−2^, 10^−4^, 10^−5^, 10^−6^ and 10^−9^ mol L^−1^ (M), respectively, under the laser excitation of 532 nm with same exposure time of 10 s. For contrast, the Raman spectra intensity of BPE on bare silicon was multiplied by 10. The enhancement factor (EF) is defined by EF = (I_*SERS*_ × N_*bulk*_)/(I_*bulk*_ × N_*SERS*_)^49^, where I_*sers*_ is the Raman intensity of the 1200 cm^1^ band resulting from BPE molecules on the Ag dot arrays substrate and I_*bulk*_ is the Raman signal of the same band on the bare Si substrate. N_*SERS*_ and N_*bulk*_ are the numbers of molecules on the illuminated area, which are proportional to the effective surface area of the pattern. According to the equation, the EF is estimated to 1.64 × 10^8^. Besides, the Raman signal of very low concentration of BPE, such as 10^−9^ M shown in Fig. 8a, can be detected through the as-prepared Ag nanodots substrate.

Figure 8b shows the reproducibility for Raman signal of BPE molecules with concentration of 10^−4^ M at ten random different points on pre-fabricated 2 × 2 cm^2^ SERS-active substrate. The variable coefficient of the intensity of Raman signal at 1200 cm^−1^ was measured to be less than 5%, which benefits from the uniformity of Ag nanodot arrays attributed to the phase separation lithography that we developed.

## Discussion

In summary, we proposed a maskless lithographic approach based on phase separation of PS and PEG immiscible polymer blend system through spin-coating process. The process of fabricating a monolayer of porous nanostructure based on this phase separation system were demonstrated, and the required feature size of porous nanostructure could be obtained conveniently by tuning the parameters of PS/PEG solution system and the spin-coating speed. As an example, a typical PS nanopore structure with a diameter mainly range from 200 to 400 nm was fabricated through this method. Combining this new strategy of lithography based on phase separation of polymer blends with conventional nanofabrication methods, such as RIE, PVD and lift-off processes, as an example of applications, we fabricated wafer-scaled silicon-based AR (total absorption) nanostructure with a broad bandwidth for photovoltaic applications, which shows a polarization-independence and slight dispersion for the incident angles. In addition, Ag nanodot arrays with large-area on silicon substrate were fabricated and exhibited a SERS enhancement factor of up to 1.64 × 10^8^ with high uniformity, which might be potential for practical use in Raman-based biological and chemical sensing devices. Compared with traditional fabrication methods, the phase separation lithography is an extremely simple, low-cost, and easily accessible methods for fabrication of nanostructure with wafer-scale.

## Methods

### Materials

All the polymer materials including PS (Mw = 100,000), PEG (Mn = 526), acetone, toluene and other reagents are commercially available and used without further purification. Trans-1,2-bis(4-pyridyl)ethylene (BPE) was purchased from Sigma-Aldrich Co. The silicon wafer is P type with the orientation of (100). The standard RCA cleaning and ultrasonic cleaning in acetone and ethanol were carried out before the wafer was used.

### Preparation of PS nanopore structures

Polymer blend solutions with various PS/PEG weight ratios and concentrations were prepared by dissolving each mixture of PS and PEG in toluene (concentration expressed as % w/w). The blend film was formed by spin coating the solutions on a silicon substrate. Then the substrate was dipped into DI water to remove PEG moiety for less than 5 seconds and blow dried with nitrogen gas.

### Preparation of surface-modified silicon for AR

After the PS nanopore structures on silicon substrate was prepared by the phase separation lithography, the residual PS layer under the nanopores was removed by RIE with O_2_ gas flow rate of 10 sccm, process pressure of 2 Pa and RF power of 40 W (RIE 100, Oxford). A 10 nm Ni was deposited on the etched film by a vacuum e-beam evaporation. Ni nanodot arrays with a thickness of 10 nm were formed by lift-off process in chlorobenzene. Taking the nickel nanodot arrays as etching mask, the silicon substrate was etched by CHF_3_ RIE with flow rate of 20 sccm, process pressure of 2 Pa and RF power of 50 W.

### Preparation of Ag nanodot arrays

Ag layer with a thickness of 65 nm was deposited on the residual-layer-removed nanoporous film by a vacuum e-beam evaporation. Ag nanodot arrays were obtained after a lift-off process in chlorobenzene.

### Characterization

All the SEM images were detected using field-emission scanning electron microscope (ZEISS ULTRA-55). The reflectance of AR wafer and bare silicon wafer was obtained by an UV-VIS spectrophotometer with an integrating sphere at near-normal incident angle of 8^o^ (Shamrock SR303i, Andor Technology). The SERS signals were recorded using an upright confocal Raman microscope (Labram Aramis Raman Spectrometer, Horiba Scientific) equipped with a nitrogen-cooled multichannel CCD detector and through a 50 × objective. 532 nm wavelength laser was used with exposure time of 10 seconds for BPE. The power of excitation laser at the sample was ~0.5 mW and the spot size was about 2 μm^2^.

## Additional Information

**How to cite this article**: Guo, X. *et al.* A New Strategy of Lithography Based on Phase Separation of Polymer Blends. *Sci. Rep.* **5**, 15947; doi: 10.1038/srep15947 (2015).

## Supplementary Material

Supplementary Information

## Figures and Tables

**Table 1:** Summarization of feature size distribution of PS nanopore structures with various PS/PEG ratio, spin speed and concentration.

PS/PEG weight ratio	Spin speed [r∙min^−1^]	Concentration [%]	Average feature size [nm]	Standard deviation [nm]	Feature density [features inch^−2^]
1:2	4000	5	374.4	77	2.69 × 10^9^
1:2.5	4000	5	489.3	171	1.10 × 10^9^
1:3	4000	5	603.1	515	5.38 × 10^8^
1:4	4000	5	666.0	1076	4.57 × 10^8^
1:2	3000	5	382.1	95	2.97 × 10^9^
1:2	5000	5	307.2	73	2.87 × 10^9^
2:3	3000	5	267.9	61	4.93 × 10^9^
2:3	3000	4	250.9	45	6.06 × 10^9^
2:3	3000	3	105.4	27	8.48 × 10^9^
2:3	3000	2	—	—	—
