# Supplementary material for: A New Strategy of Lithography Based on Phase Separation of Polymer Blends
Source: Sci Rep. 2015 Oct 30;5:15947. doi: 10.1038/srep15947 (PMC4626759; doi:10.1038/srep15947)
Supplement: Supplementary Information [file srep15947-s1.pdf]

Supporting Information

A New Strategy of Lithography Based on Phase Separation of Polymer Blends

Xu Guo,[1,3] Long Liu,[2,3] Zhe Zhuang,[3,4] Xin Chen,[5] Mengyang Ni,[1,3] Yang Li,[1,3] Yushuang Cui,[1,3] Peng Zhan,[2,3,6,*] Changsheng Yuan,[1,3] Haixiong Ge,[1,3,6,*] Zhenlin Wang,[2,3,6] and Yanfeng Chen[1,3,6]

[1]Department of Materials Science and Engineering, College of Engineering and Applied Sciences, Nanjing University, Nanjing, 210093, China

[2]Department of Physics, Nanjing University, Nanjing, 210093, China

[3]National Laboratory of Solid State Microstructures, Nanjing, 210093, China

[4]Jiangsu Provincial Key Laboratory of Advanced Photonic and Electronic Materials, School of Electronic Science and Engineering, Nanjing University, Nanjing, 210093, China

[5]Department of Polymer Science & Engineering and Key Laboratory of High Performance Polymer Materials & Technology of MOE, School of Chemistry & Chemical Engineering, Nanjing University, Nanjing, 210093, China

[6]Collaborative Innovation Center of Advanced Microstructures, Nanjing 210093, China

*e-mail: haixiong@nju.edu.cn, zhanpeng@nju.edu.cn,

Figure S1 presents the feature size distribution of PS nanopore structures with different PS/PEG weight ratios. Obviously, the uniformity of pore size decreased sharply with the increase of PEG in the blend. With PS/PEG=1:2, the pore size was

mainly under 500 nm, as shown in Figure S1a. The pore size ranged from 170 to 1200 nm when the PS/PEG weight ratio was 1:2.5, shown in Figure S1b. When the PS/PEG weight ratios were 1:3 and 1:4, the pore size ranged from 190 to 2600 nm and 150 to 5000 nm, as shown in Figure S1c and d, respectively.

Figure S2 shows the feature size distribution of PS nanopore structures with various concentrations of (a) 5%, (b) 4% and (c) 3%, while the PS/PEG weight ratio and spin speed were set at 2:3 and 3000 r min^{-1}, respectively. With the concentration decreased, the pore size decreased. While the concentration was 5%, 4% and 3%, the pore size ranged from 59 to 385 nm, 96 to 365 nm and 26 to 166 nm, respectively.

Figure S3a to c present the SEM images of PS nanopore structures with different spin speeds (a) 3000 r min^{-1}, (b) 4000 r min^{-1} and (d) 5000 r min^{-1}, respectively. Figure S3a' to c' are the corresponding feature size distributions of PS nanopore structures. From 3000 r min^{-1} to 5000 r min^{-1}, the pore size ranged from 102 to 727 nm, 90 to 550 nm and 84 to 501 nm, as shown in Figure S3a' to c' respectively. The average pore size turned smaller with the spin speed increased.

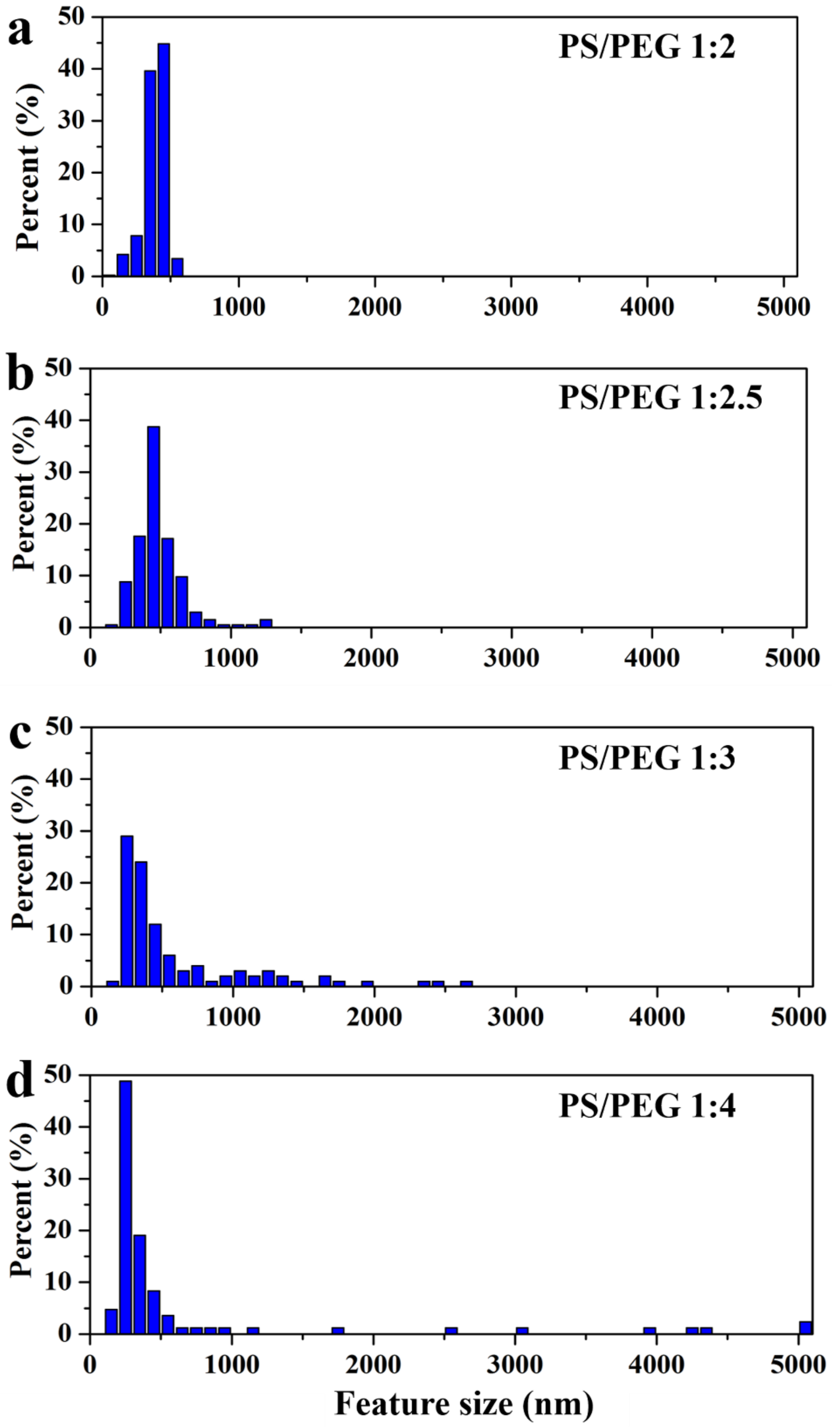

Figure S1. Feature size distribution of PS nanopore structures with different PS/PEG weight ratios. (a) PS:PEG=1:2; (b) PS:PEG=1:2.5; (c) PS:PEG=1:3; (d) PS:PEG=1:4.

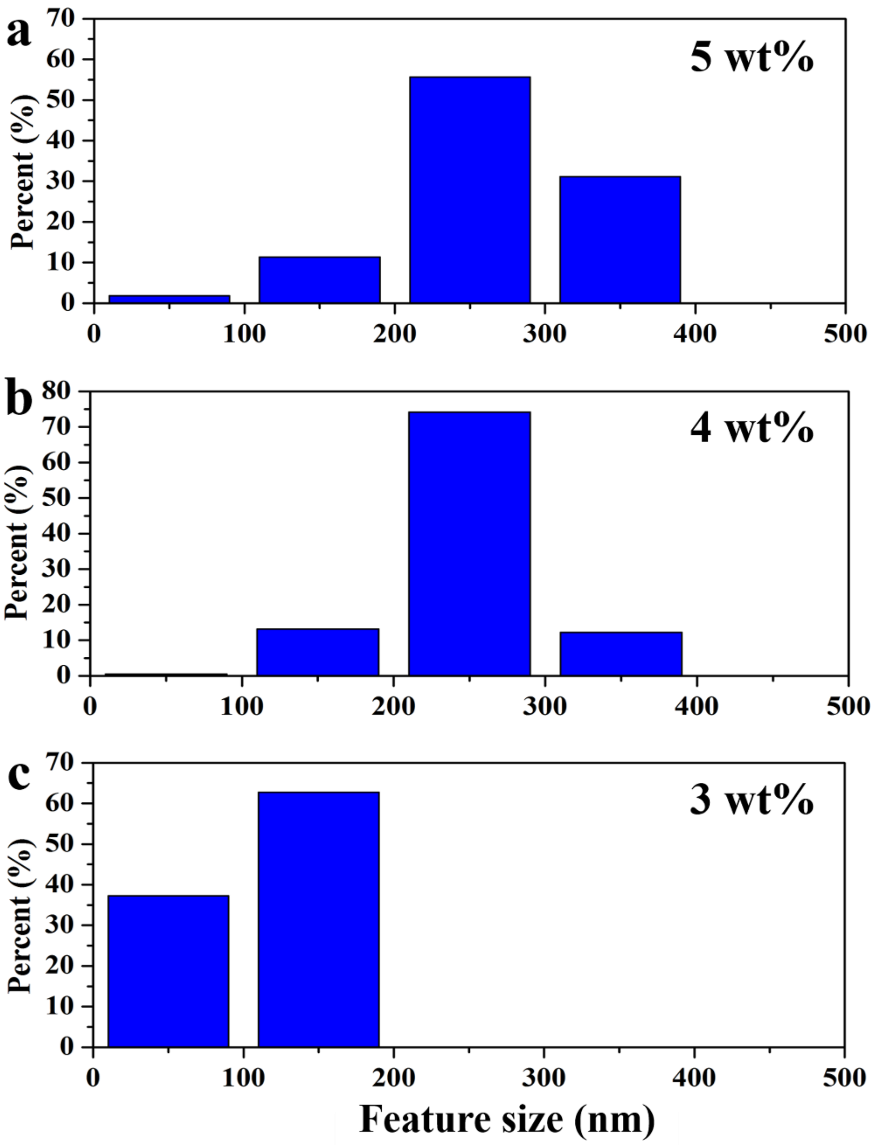

Figure S2. Feature size distribution of PS nanopore structures with different initial concentrations. (a) 5%; (b) 4%; (c) 3%. The PS/PEG weight ratio and spin speed were fixed at 2:3 and 3000 r min^{-1}, respectively.

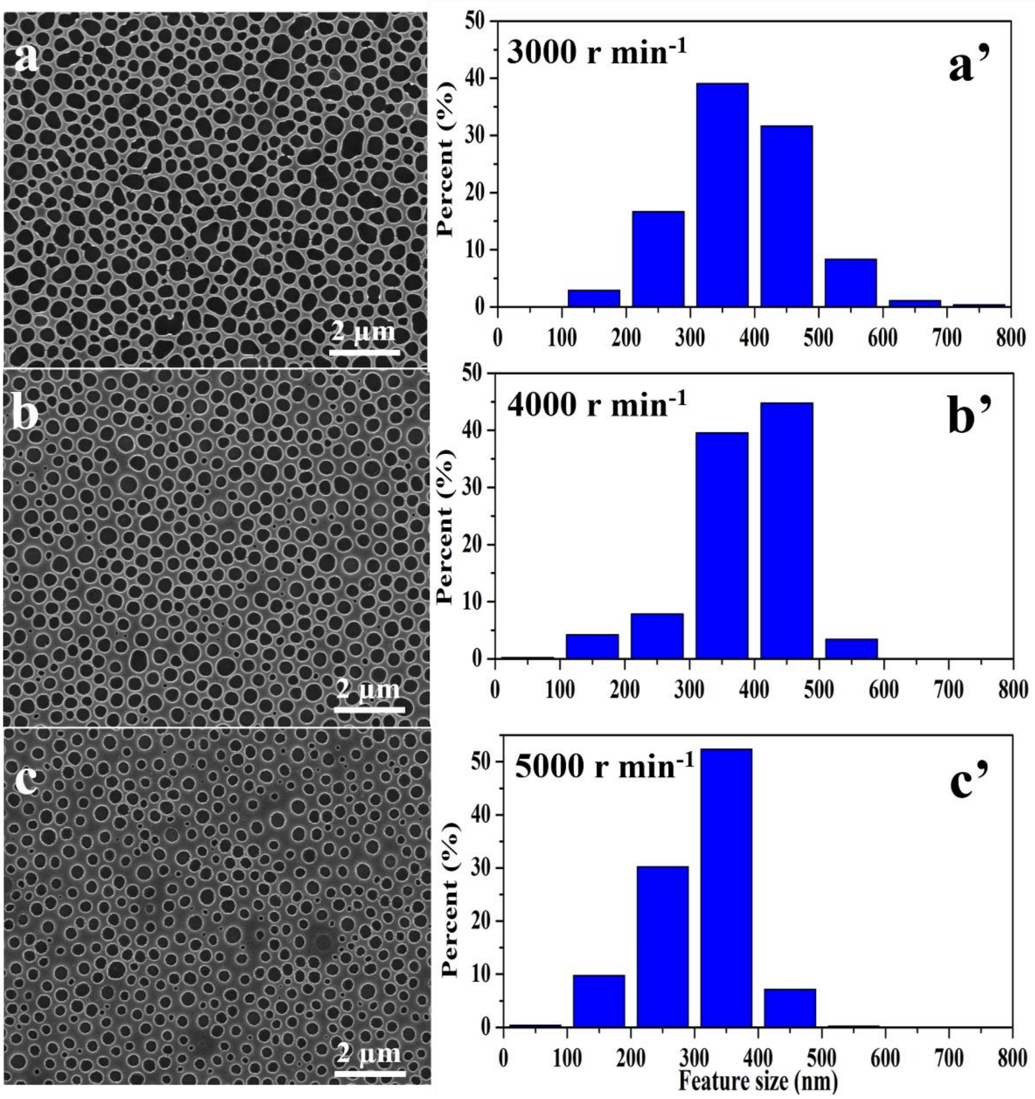

Figure S3. Top view of SEM images of PS nanopore structure produced with (a) 3000 r min^{-1}, (b) 4000 r min^{-1} and (c) 5000 r min^{-1}. (a’) (b’) and (c’) are the corresponding feature size distribution of PS nanopore structure. The PS/PEG weight ratio and concentration are fixed at 1:2 and 5%, respectively.
